# Stabilization of Nrf2 Protein by D3T Provides Protection against Ethanol-Induced Apoptosis in PC12 Cells

**DOI:** 10.1371/journal.pone.0016845

**Published:** 2011-02-03

**Authors:** Jian Dong, Dong Yan, Shao-yu Chen

**Affiliations:** 1 Bowles Center for Alcohol Studies, University of North Carolina, Chapel Hill, North Carolina, United States of America; 2 Department of Cancer Biology and Pharmacology, University of Illinois College of Medicine, Peoria, Illinois, United States of America; Johns Hopkins, United States of America

## Abstract

Previous studies have demonstrated that maternal ethanol exposure induces a moderate increase in Nrf2 protein expression in mouse embryos. Pretreatment with the Nrf2 inducer, 3H-1, 2-dithiole-3-thione (D3T), significantly increases the Nrf2 protein levels and prevents apoptosis in ethanol-exposed embryos. The present study, using PC12 cells, was designed to determine whether increased Nrf2 stability is a mechanism by which D3T enhances Nrf2 activation and subsequent antioxidant protection. Ethanol and D3T treatment resulted in a significant accumulation of Nrf2 protein in PC 12 cells. CHX chase analysis has shown that ethanol treatment delayed the degradation of Nrf2 protein in PC12 cells. A significantly greater decrease in Nrf2 protein degradation was observed in the cells treated with D3T alone or with both ethanol and D3T. In addition, D3T treatment significantly reduced ethanol-induced apoptosis. These results demonstrate that the stabilization of Nrf2 protein by D3T confers protection against ethanol-induced apoptosis.

## Introduction

Maternal consumption of alcohol during pregnancy results in a range of structural and functional birth defects. Fetal Alcohol Spectrum Disorders (FASD) is an umbrella term describing the range of effects that can occur in an individual whose mother drank alcohol during pregnancy. Prenatal alcohol exposure is considered to be the leading known non-genetic cause of mental retardation in the Western world [1], [2].

Studies using both rodent and avian animal models have shown that the vulnerability of selected cell populations to ethanol-induced apoptosis is a major component in the pathogenesis of ethanol-induced malformations [3]–[8]. Excessive cell death was found in specific regions of the brain of gestational day 8.5 to 9 ethanol-exposed mouse embryos [6], [7]. Among the vulnerable cell populations are cranial neural crest cells (NCCs) [3], [4], [7], [9]. In addition, ethanol exposure during the period of brain development resulted in the death of neurons in the hypothalamus [10], cerebral cortex [11], cerebellum [12], and associated brain–stem structures [13]. The ethanol-induced cell death in embryos has been shown to be apoptotic [5], [6], [11], [14], [15].

Among a number of mechanisms underlying ethanol-induced apoptosis and subsequent malformations is oxidative stress [4], [5], [15]–[19]. Reactive oxygen species (ROS) generation has been observed in mouse embryos exposed to ethanol both *in vitro* and *in vivo* [5], [16], [18]. Antioxidants have been shown to diminish ethanol-induced superoxide anion generation, lipid peroxidation and cell death, as well as the incidence of neural tube defects in cultured mouse embryos [18]. *In vivo* studies have also shown that ethanol-induced apoptosis in selected cell populations in the developing limb buds and subsequent limb defects can be partially prevented by maternal treatment with an SOD and catalase mimetic, EUK-134 [15]. However, while antioxidants are promising for human application, the incomplete protection provided by exogenous antioxidants points to the limitations associated with the use of exogenous antioxidants. Therefore, a strategy for protecting against oxidative injury through the upregulation of endogenous antioxidants holds significant promise for the prevention of FASD.

Recently, Nrf2 has been demonstrated to be a key transcription factor that regulates the induction of antioxidant genes [20], [21]. Under basal conditions, Nrf2 is mainly in the cytoplasm through binding to Kelch-like ECH-associated protein 1 (Keap1), which in turn facilitates the ubiquitylation and subsequent proteolysis of Nrf2 in a constitutive manner [22]–[25]. In response to oxidative stress, Nrf2 dissociates from Keap1, translocates into the nucleus and elicits the antioxidant response by induction of a battery of gene products, including antioxidant and phase II detoxification enzymes [26], [27]. It has been suggested that activation of Nrf2 is dependent on the mechanisms that increase its stability [28], [29].

There are a numbers of natural and synthetic small molecules that can induce Nrf2 activation [30]–[32]. Among these potent Nrf2 inducers are isothiocyanates, tert-butylhydroquinone (tBHQ) and 1,2-dithiole-3-thiones (D3T) [32]–[34]. D3T is a potent cancer chemopreventive agent that prevents against mutation and initiation of neoplasia [35]. Activation of the Nrf2 pathway by oral administration of D3T has also been reported to confer partial protection against MPTP-induced neurotoxicity [36]. The protective effects of D3T have been associated with induction of the detoxifying and antioxidant enzymes SOD, catalase and γ -glutamylcysteine synthetase (γ - GCS) [32], [35], [37].

A recent study, using an *in vivo* mouse model, has shown that ethanol treatment resulted in increased Nrf2 protein levels and Nrf2-ARE binding in mouse embryos. Maternal ethanol exposure also resulted in a moderate increase in the mRNA and protein expression of Nrf2 downstream target antioxidant genes. Pretreatment with D3T significantly increased Nrf2 protein levels and Nrf2-ARE binding, and strongly induced the mRNA and protein expression of Nrf2 downstream target genes. In addition, D3T pretreatment resulted in a significant decrease in ROS generation and apoptosis in ethanol-exposed embryos [16]. These results support the hypothesis that Nrf2 signaling is involved in induction of an antioxidant response in ethanol-exposed mouse embryos.

Although the *in vivo* study using intact embryos has clearly demonstrated that D3T can induce Nrf2 activation and prevent ethanol-induced oxidative stress and apoptosis, the mechanisms by which D3T enhances Nrf2-mediated transcriptional activation and subsequent antioxidant protection remain elusive. Using PC12 cells, a cell line derived from the neural crest, as an *in vitro* model, the current study was designed to elucidate the mechanisms underlying D3T mediated Nrf2 activation and subsequent antioxidant protection in ethanol-exposed neural crest cells and other developing neuronal cells. The results of this study support the hypothesis that the stabilization of Nrf2 protein by D3T is one of the important mechanisms underlying D3T-mediated Nrf2 activation and antioxidant protection against ethanol-induced apoptosis in PC12 cells.

## Results

### Ethanol exposure and D3T treatment significantly increased Nrf2 protein expression in PC12 cells

**Figure 1:**
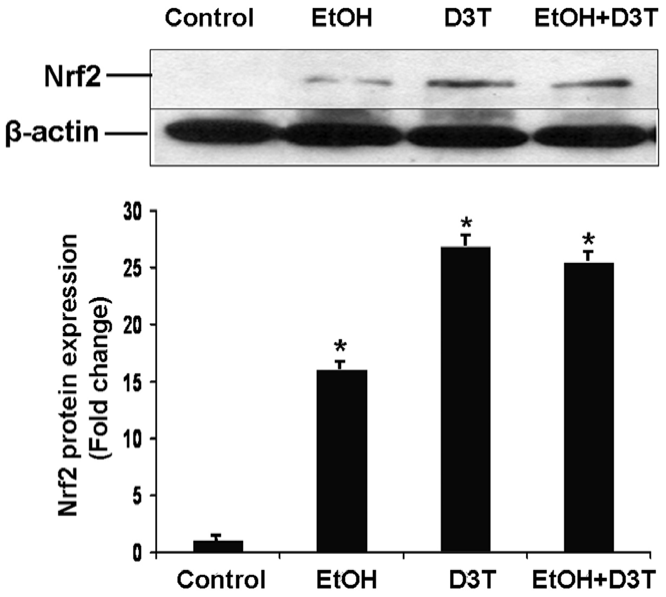
Treatment with ethanol and D3T increased the level of Nrf2 protein in PC12 cells. Western blot was performed to analyze the level of Nrf2 protein in PC12 cells. Cell lysates were prepared from PC12 cells cultured in control medium (Control), treated with 200 mM ethanol (EtOH), treated with 50 µM D3T alone (D3T), or treated with both ethanol and D3T (EtOH+D3T). Data are expressed as fold change over control and represent the mean ± SEM of three separate experiments. * *p<*0.05 *vs.* control.

To determine whether ethanol exposure can increase the Nrf2 protein levels, PC 12 cells were treated with 200 mM ethanol for 48 hours and the Nrf2 protein level was determined by western blot. As shown in Fig. 1, ethanol exposure resulted in a 15-fold increase in the Nrf2 protein levels. To examine the effects of D3T on Nrf2 protein expression, PC 12 cells were pretreated with 50 µM D3T for 16 hours, following concurrent exposure to D3T and ethanol for 48 hours. Treatment of PC12 cells with D3T alone showed a 25-fold increase in Nrf2 protein levels as compared to the control group. The combination of the D3T treatment and ethanol exposure yielded an increase in Nrf2 protein expression that is comparable to that in the cells treated with D3T alone. These results indicate that while both ethanol and D3T can increase Nrf2 protein expression, D3T treatment resulted in a significantly greater increase in Nrf2 levels in control and ethanol-exposed PC 12 cells.

### Treatment with ethanol and D3T delayed the degradation of Nrf2 protein in PC12 cells

**Figure 2:**
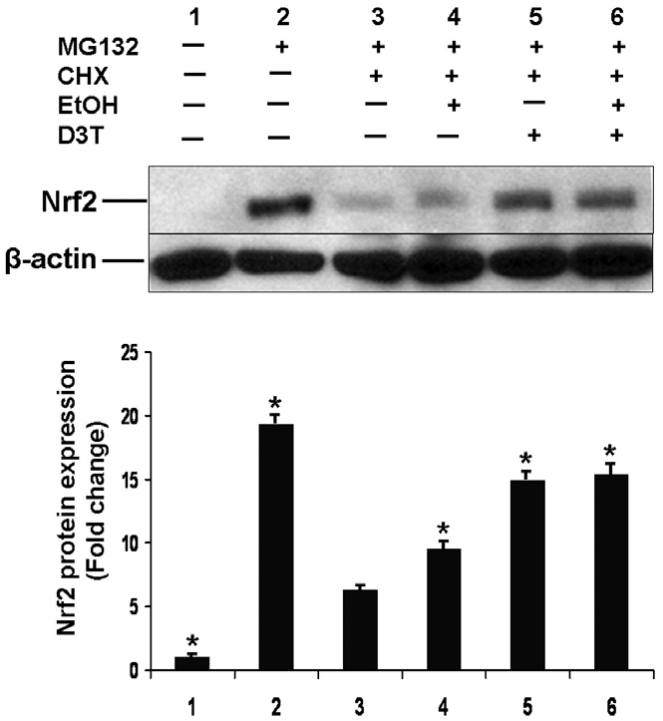
CHX chase analysis showed that treatment with ethanol and D3T delayed the degradation of Nrf2 protein in PC12 cells. Western blot was performed to analyze the level of Nrf2 protein in PC12 cells. Cell lysates were prepared from PC12 cells cultured in control medium (lane 1), treated with proteasome inhibitor MG132 (30 µM) for 3 hrs (lane 2), treated with MG132 for 3 hrs, followed by treatment for 2 hrs with CHX (an inhibitor of protein synthesis,100 µg/ml) alone (lane 3), or CHX along with either 200 mM ethanol (lane 4), 50 µM D3T (lane 5), or ethanol plus D3T (lane 6). Data are expressed as fold change over control and represent the mean ± SEM of three separate experiments. * *p<*0.05 *vs.* group treated with MG132 and CHX (lane 3).

To determine the effects of ethanol and D3T on Nrf2 protein stability, CHX chase analysis was performed in PC 12 cells. Cells were pretreated with the proteasome inhibitor, MG132 for 3 hours to inhibit the proteasomal degradation of Nrf2. The cells were then treated for 2 hours with CHX alone or along with either ethanol, D3T, or ethanol plus D3T. A significant increase in Nrf2 protein expression was observed in MG132-treated PC 12 cells, suggesting that Nrf2 protein is degraded by the proteasome in PC12 cells. CHX chase analysis has shown that ethanol treatment delayed the degradation of Nrf2 protein in PC12 cells. A significantly greater decrease in Nrf2 protein degradation was observed in PC 12 cells treated with D3T alone or co-treated with both ethanol and D3T (Fig. 2), indicating that D3T can significantly increase Nrf2 protein stability in PC12 cells.

### D3T treatment significantly prevented apoptosis in ethanol-exposed PC12 cells

**Figure 3:**
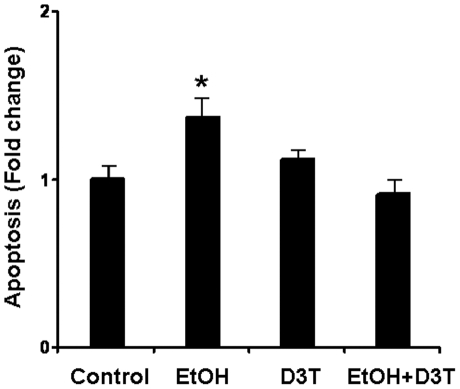
Flow cytometry of apoptotic PC12 cells with Annexin V-FITC showed that D3T treatment significantly prevents ethanol-induced apoptosis. Apoptosis was measured in PC12 cells cultured in control medium (Control), treated with 200 mM ethanol (EtOH), treated with 50 µM D3T alone (D3T), or treated with both ethanol and D3T (EtOH+D3T). Data are expressed as fold change over control and represent the Mean ± SEM of three separate experiments. *p<0.05 *vs.* control. #*p<*0.05 *vs.* EtOH.

In recognizing that excessive apoptosis is a prominent pathogenic feature in FASD models and that D3T induced Nrf2 activation can prevent apoptosis in mouse embryos exposed to ethanol, the potential of D3T to diminish apoptosis in ethanol-exposed PC12 cells was tested. Flow cytometric analysis revealed a significant increase in apoptosis in the ethanol-exposed PC12 cells as compared to the control cells. D3T treatment reduced the apoptosis in ethanol-exposed PC12 cells to the level comparable to that in control cells (Fig. 3).

## Discussion

The vulnerability of selected cell populations, including neural crest cells (NCCs), to ethanol-induced cytotoxicity is one of the important factors that contribute to the genesis of alcohol-related birth defects. NCCs are progenitors of multiple cell types, including the skeletal and connective tissues of the face [38]–[40]. Ethanol has been shown to induce apoptotic cell death in NCCs, a result that appears to contribute heavily to subsequent abnormalities [3], [4], [7], [41]. To elucidate the mechanisms underlying D3T-mediated Nrf2 activation and subsequent protection against ethanol-induced apoptosis in neural crest cells and other developing neuronal cells, PC12 cells, a cultured rat pheochromocytoma cell line, was used as an *in vitro* model. PC12 cells appear to be a good model system to current study because these cells are derived from the neural crest [42] and are commonly utilized in the studies regarding the molecular and cellular mechanisms of neuronal development. In addition, PC12 cells have been used extensively to study the mechanisms underlying ethanol-induced cell death [43]–[45]. In the present study, flow cytometric analysis using an Annexin V-FITC Apoptosis Detection Kit showed significantly increased apoptosis in ethanol-exposed PC12 cells. These findings are consistent with previous reports illustrating ethanol-induced cell death in PC12 cells [43]–[45].

The role of Nrf2 in cell survival has been supported by a number of studies. Fas-induced apoptosis has been shown to be prevented by over-expression of Nrf2 [46]. Activation of Nrf2 was also found to protect against nitric oxide-induced apoptosis [47] as well as cell death in an *in vitro* model of ischemia/reperfusion [48]. Additionally, in response to elevated chromium (VI) and cadmium, increased ROS production and apoptosis were observed in mouse embryonic fibroblast cells lacking Nrf2 [49], [50]. In this study, a chemical inducer of Nrf2, D3T, has been shown to potently increase Nrf2 levels and significantly diminish apoptosis in ethanol-exposed PC12 cells. These results are consistent with a previous *in vivo* study showing that D3T can diminish ethanol-induced apoptosis in early mouse embryos by induction of Nrf2 [16]. The results also clearly demonstrate that D3T is a potent Nrf2 inducer that can induce an antioxidant response and prevent ethanol-induced apoptosis. However, in this study, an increase in Nrf2 levels was also observed in PC12 cells exposed to ethanol alone. The observed Nrf2 activation in PC12 cells exposed to ethanol alone was expected because exposure of cells to ethanol results in the generation of ROS [51], which are known to activate Nrf2 [26]. This response is not unique to ethanol-exposed cells. Increases in Nrf2 levels have also been observed in cells treated with a numbers of other toxic chemicals, including heavy metals [49], [52] and cigarette smoke [53], as well as in the livers and hepatocytes of alcohol-fed mice and rats [54]. The fact that excessive apoptosis results from the same ethanol exposure regimen that increased Nrf2 levels suggests that the ethanol-induced activation of Nrf2 is an adaptive response that is insufficient to be protective. This is supported by the results from the previous *in vivo* and *in vitro* studies showing that ethanol can induce only a moderate increase in Nrf2 downstream antioxidant and detoxifying gene expression [16], [51].

Nrf2 is a highly unstable protein and its half-life is about 15 min in untreated cells [20]. A well established mechanism that controls Nrf2 activation is that oxidative stress or Nrf2 inducers can increase Nrf2 protein stability, resulting in its accumulation in the cells [28], [29]. The Keap1-Nrf2 complex appears to play critical role in facilitating the degradation of Nrf2 [55]–[57]. It has been suggested that ubiquitination of the cytoplasmic Nrf2 involves the Keap1-Cul3-dependent E3. Keap1 functions as a BTB-containing substrate adaptor protein for Cul3 and brings Nrf2 into the Cul3-Rbx1 complex for ubiquitination [55]–[57]. However, the molecular mechanisms underlying the stabilization of Nrf2 protein by various exogenous Nrf2 inducers have not been clearly addressed.

Although D3T has long been of interest as an effective inducer of Nrf2, the mechanisms underlying D3T mediated Nrf2 activation are not fully understood. Studies have suggested that the interaction between D3T and the sulfhydryl groups of Keap1 can cause dissociation of Keap1 from Nrf2, leading to Nrf2 activation [58]. In addition, mitogen-activated protein kinases (MAPKs) have recently been shown to be involved in the activation of Nrf2 by D3T [59]. In this study, a significant increase in Nrf2 protein levels was observed in MG132-treated PC12 cells, suggesting that Nrf2 protein is degraded by the proteasome in the cells. CHX chase analysis has shown that ethanol treatment delayed the degradation of Nrf2 protein in PC12 cells. A significantly greater decrease in Nrf2 protein degradation was observed in PC12 cells treated with D3T alone or co-treated with both ethanol and D3T. These results suggest that D3T induces Nrf2 activation in PC12 cells by enhancing Nrf2 stability, resulting in elevated Nrf2 levels, and ultimately induction of Nrf2-ARE-dependent gene transcription.

In conclusion, the results of the current study demonstrate that increased Nrf2 protein stability is a mechanism underlying D3T-mediated Nrf2 activation, ultimately leading to an enhanced protection against ethanol-induced apoptosis in PC12 cells. These results, along with the findings from a previous study showing that D3T can induce an antioxidant response and prevent ethanol-induced apoptosis in early mouse embryos by induction of Nrf2, support the potential of Nrf2 inducers in attenuating oxidative tissue damage and in conferring *in vivo* protection against a variety of ROS related disease and disorders, including FASD.

## Materials and Methods

### Cell Culture and Treatments

PC12 cells (American Type Culture Collection, Rockville, MD, USA) were cultured in Dulbecco's modified Eagle's medium (DMEM) (Invitrogen, Carlsbad, CA, USA) supplemented with 10% horse serum, 5% fetal bovine serum (FBS), 100 kU/L of penicillin, and 100 mg/L of streptomycin (Sigma, St. Louis, MS, USA) at 37°C with a 5% CO2 atmosphere in a humidified incubator. For D3T pretreatment, PC 12 cells were treated with 50 µM D3T alone for 16 hours, followed by 48 hours of concurrent exposure to D3T and 200 mM ethanol. For ethanol treatment, the cells were cultured in medium containing 200 mM ethanol for 48 hours. Stable ethanol levels were maintained by placing the cell culture plates in a plastic desiccator containing 300 ml of 200 mM ethanol in distilled water. The ethanol concentration in the medium was measured using Analox Alcohol Analyzer (Model AM1, Analox Instruments USA Inc, Lunenburg, MA, USA).

### Protein Degradation Assay

Nrf2 protein degradation was analyzed by CHX-chase analysis. The PC12 cells were pretreated with proteasome inhibitor MG132 (30 µM) for 3 hours to initially inhibit the proteosomal degradation of Nrf2, leading to the accumulation of Nrf2 for degradation studies. Following extensive washing with fresh media to remove the inhibitor, the cells will then be treated for 2 hours with cycloheximide (CHX, an inhibitor for protein synthesis, 100 µg/ml) alone or along with either 200 mM ethanol, 50 µM D3T, or ethanol plus D3T. Nrf2 protein levels were analyzed by Western blot.

### Western Blotting

PC12 cells were washed in phosphate-buffered saline (PBS) and lysed for 30 min in RIPA lysis buffer (PBS, 0.5% Sodium Deoxycholate, 1% NP-40, 0.1% SDS, 1 mM Dithiothreitol) with 1 mM PMSF and protease cocktail inhibitors (Roche, Applied Science, Indianapolis, IN, USA). The samples were then centrifuged at 16,000 g for 10 min at 4°C. The supernatants were collected for Western blotting. Western blots were performed by standard protocols as described previously [5]. Briefly, the protein was resolved on an SDS-10% polyacrylamide gel and transferred to nitrocellulose membranes. The levels of Nrf2 were analyzed with rabbit polyclonal anti-Nrf2 IgG (1∶500, Santa Cruz, Santa Cruz, CA, USA), followed by detection with ECL plus western blotting detection reagents (GE Healthcare, Piscataway, NJ, USA). The membranes were then scanned on a Bio-Rad Versa DocTM Imaging System (Model 4000) and the intensity of the protein bands was analyzed using the Bio-Rad Quantity One software (Version 4.5.1).

### Flow-Cytometric Analysis of Apoptosis

Analysis of apoptosis was performed using an Annexin V-FITC Apoptosis Detection Kit (BD Bioscience, San Jose, CA, USA) according to the manufacturer's instructions. PC12 cells were washed twice with PBS and then resuspended in 1X binding buffer at a concentration of 1×10^6^ cells/ml. Then 100 µl of the solution (1×10^5^ cells) was transferred to a 5 ml tube and 5 µl of Annexin V was added into the tube. The cells were incubated for 15 min at room temperature in the dark and then analyzed in a Dako CyAn flow cytometer (Beckman-Coulter Dako CyAn ADP).

### Statistical Analysis

Statistical analyses were performed using StatView software (SAS Institute Inc, Cary, NC, USA). Data are expressed as mean ± SEM of three separate experiments. Statistical comparisons between groups were analyzed by a One-way ANOVA. Multiple comparison post-tests between groups were conducted by using Bonferroni's test. Differences between groups were considered significant at *p*<0.05.
